# Strategies for Enhancement of Live-Attenuated *Salmonella*-Based Carrier Vaccine Immunogenicity

**DOI:** 10.3390/vaccines9020162

**Published:** 2021-02-17

**Authors:** James E. Galen, Rezwanul Wahid, Amanda D. Buskirk

**Affiliations:** 1 Center for Vaccine Development and Global Health, University of Maryland School of Medicine, Baltimore, MD 21201, USA; rwahid@som.umaryland.edu; 2 Center for Drug Evaluation and Research, Office of Pharmaceutical Quality, Office of Process and Facilities, Division of Microbiology Assessment II, U.S. Food and Drug Administration, Silver Spring, MD 20903, USA; abuskirk02@gmail.com

**Keywords:** Salmonella, Typhi, carrier vaccine, immunogenicity, homeostasis, inflammation

## Abstract

The use of live-attenuated bacterial vaccines as carriers for the mucosal delivery of foreign antigens to stimulate the mucosal immune system was first proposed over three decades ago. This novel strategy aimed to induce immunity against at least two distinct pathogens using a single bivalent carrier vaccine. It was first tested using a live-attenuated *Salmonella enterica* serovar Typhi strain in clinical trials in 1984, with excellent humoral immune responses against the carrier strain but only modest responses elicited against the foreign antigen. Since then, clinical trials with additional *Salmonella*-based carrier vaccines have been conducted. As with the original trial, only modest foreign antigen-specific immunity was achieved in most cases, despite the incorporation of incremental improvements in antigen expression technologies and carrier design over the years. In this review, we will attempt to deconstruct carrier vaccine immunogenicity in humans by examining the basis of bacterial immunity in the human gastrointestinal tract and how the gut detects and responds to pathogens versus benign commensal organisms. Carrier vaccine design will then be explored to determine the feasibility of retaining as many characteristics of a pathogen as possible to elicit robust carrier and foreign antigen-specific immunity, while avoiding over-stimulation of unacceptably reactogenic inflammatory responses.

## 1. Introduction

The concept of bacterial-based carrier vaccines rests on the notion that an attenuated strain of bacteria, genetically engineered to be safe yet still immunogenic, can be further engineered by the introduction of additional genes encoding protective antigens from unrelated pathogens; the resulting multivalent candidate vaccine should then be capable of eliciting biologically relevant protective immune responses against both the carrier vaccine itself as well as the additional target pathogens. Although simple in principle and elegant in terms vaccine development, this novel approach has required refinement through various iterations over the last three decades, with testing in clinical trials yielding only limited success. In this review, we will re-examine the engineering of *Salmonella enterica*-based carrier vaccines with an eye towards optimizing protective efficacy while maintaining acceptable reactogenicity. We will first summarize the state-of-the-art for clinical trials conducted using carrier vaccines derived from *S.* Typhi. We will then attempt to deconstruct the lack of carrier vaccine immunogenicity in humans by examining the basis of bacterial immunity in the human gastrointestinal tract and how the gut detects and responds to pathogens. Carrier vaccine design will then be explored to determine the feasibility of retaining as many characteristics of a pathogen as possible to elicit robust carrier and foreign-antigen-specific immunity, while avoiding overstimulation of unacceptably reactogenic inflammatory responses. The insights gleaned from this discussion will provide a basis for possible solutions that may significantly improve the overall immunogenicity of *S.* Typhi-based carrier vaccines in humans.

## 2. A summary of *S.* Typhi-based Carrier Vaccine Performance in Clinical Trials

Since 1984, at least a dozen *S.* Typhi-based carrier vaccines have been tested in clinical trials, as summarized in Table 1. In general, these vaccines were derived from the fully virulent clinical isolate Ty2, which was attenuated by chromosomal deletions affecting critical metabolic pathways, as opposed to deletion(s) of chromosomally encoded virulence factors. The carrier strains delivered only a single foreign antigen, which was typically of prokaryotic origin and targeted pathogenic bacteria. In most cases, the foreign antigen was expressed from a multicopy plasmid that did not encode resistance to an antibiotic (typically included in plasmids for selection purposes, but currently discouraged from use in licensed human oral vaccines by the Food and Drug Administration). To ensure retention in vivo within carrier vaccines after mucosal immunization, most expression plasmids also encoded a balanced lethal system in which loss of the plasmid would result in lysis of the carrier vaccine. This prevents plasmid-less organisms from outgrowing carrier organisms and tilting adaptive immune responses away from the foreign antigen and towards the carrier strain itself. Where expression plasmids were not used, foreign-antigen-encoding gene cassettes were stably integrated into the chromosome, again assuring stability of expression while avoiding the use of antibiotics to select for expression. All volunteers in these trials were immunized mucosally, with most immunized orally and only one cohort receiving rectal immunization [1]. In general, the immunization strategy involved delivery only with the carrier strain, although vaccination by a heterologous prime–boost regime, in which volunteers first received the carrier strain orally and were then boosted intramuscularly with adjuvanted foreign antigen, has also been conducted.

Most reported clinical trials studying *S.* Typhi-based carrier vaccines have focused on foreign-antigen-specific humoral immunity rather than antigen-specific, cell-mediated immunity. However, few of these trials demonstrated foreign-antigen-specific seroconversions in the majority of volunteers, with many studies reporting percentages well below 25%. A successful clinical trial described by Bumann et al. in 2010 [12] illustrates many of the study design parameters summarized above. In this study, volunteers were primed with a single oral dose of attenuated CVD 908 carrier vaccine presenting a plasmid-encoded outer membrane protein fusion of two antigens from *Pseudomonas aeruginosa*; all volunteers were then boosted intramuscularly (i.m.) 4 weeks later with a single dose of alum-adjuvanted purified fusion protein. These vaccinees mounted *P. aeruginosa*-specific serum IgG responses comparable to subjects immunized with 3 i.m. doses of adjuvanted subunit vaccine alone; however, orally primed volunteers also mounted *P. aeruginosa*-specific mucosal pulmonary IgA responses that were not observed in subjects receiving only i.m. immunizations. Interestingly, in an additional cohort of volunteers vaccinated with live vector vaccines derived from the more attenuated licensed vaccine Ty21a, three oral priming doses in addition to the i.m. booster dose were required to elicit immune responses comparable to those of volunteers receiving only a single oral priming dose of CVD 908 plus i.m. boost [12]. Although this study convincingly illustrates the proof-of-principle for using carrier vaccines to immunize via the mucosa against unrelated pathogens, most human studies conducted thus far have not been as successful in eliciting both foreign-antigen-specific and carrier-specific immunity. To gain insights into mechanisms potentially influencing the immunogenicity of live oral *Salmonella*-based carrier vaccines and the antigens that they co-express, we will briefly step back to examine a broader question of how the human intestine regulates immunity against pathogens versus non-pathogenic bacteria in the gut.

## 3. Why Are Commensal Bacteria Tolerogenic?

The gastrointestinal tract supports an extensive diversity of commensal bacteria, with the highest colonization densities residing in the colon [14,15]. The steady state condition of the gastrointestinal immune system is tolerogenic, a baseline condition referred to as homeostasis, in which commensal bacteria are prevented from access to deep tissues but are not actively cleared from the gastrointestinal tract [16]. Homeostasis is beneficial to the host for several important reasons, including colonization resistance, whereby non-pathogenic normal microbiota reside in permissive niches within the gut and compete with incoming, potentially pathogenic organisms to obstruct colonization [15]. Colonization resistance against incoming pathogens is accomplished by a variety of mechanisms, including: (1) limitation of available nutrients, (2) metabolization of host bile salts into secondary bile salts with antibacterial properties, (3) secretion of antibacterial compounds such as bacteriocins and antimicrobial peptides, and (4) cell-to-cell inhibition through type 6 secretion systems (T6SS, [17]). A recent report suggests that maintenance of the phylogenetic diversity of commensal bacteria in the human gut also exerts a significant immunomodulatory effect to promote homeostasis by balancing the response to particularly immunostimulatory bacteria with other microbial species that dampen host immune responses [18].

The metabolism of resident human microflora also provides metabolites to the host that are not normally available from human metabolic activity, including the short-chain fatty acids (SCFAs) butyrate, propionate, and acetate [19]. SCFAs contribute to the maintenance of homeostasis in several ways, including lowering of gastrointestinal pH, enhancement of mucus secretion, and stimulating the upregulation of tight junctions in intestinal epithelial cells (IECs) [20]. Butyrate in particular plays an important role in homeostasis by stimulating intestinal dendritic cells to secrete IL-10 to promote an increase in tolerogenic T_reg_ cells, thereby maintaining an important balance between immunosuppressive T_reg_s and effectors such as IL-17-secreting Th17 and Th1 effector cells to regulate inflammation [20,21].

The detailed mechanisms by which humans regulate inflammation and control mucosal immune responses against commensal bacteria to preserve homeostasis are not fully understood. The process of deciphering these pathways has been likened to the old parable of blind men in a room describing an elephant; descriptions will depend on where and what one is attempting to examine [21]. However, considerable progress has been made in recent years examining the role that various intestinal cells play in the regulation of this pivotal process. It is now well recognized that unregulated inflammatory and adaptive responses can lead to severe intestinal pathologies, including inflammatory bowel disease. Therefore, the human host ensures proper regulation of homeostasis on multiple physical and immunological levels.

**Figure 1:**
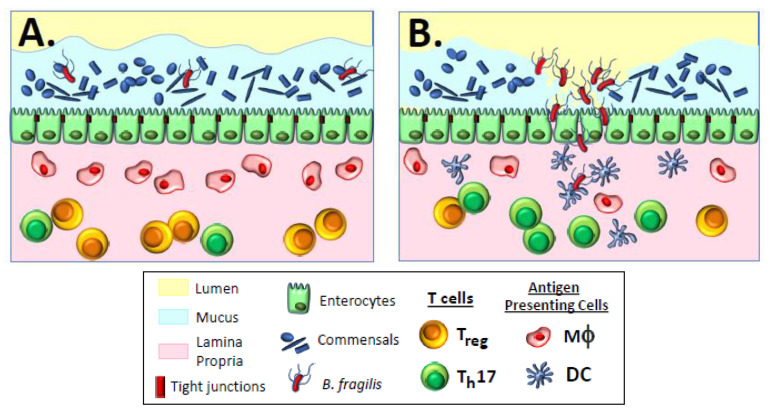
Homeostasis and inflammation in the gastrointestinal tract. (**A**) The steady state condition of the gastrointestinal immune system is tolerogenic, a baseline condition referred to as homeostasis, in which commensal bacteria are prevented from access to deep tissues but are not actively cleared from the gastrointestinal tract. During homeostasis, resident M2 macrophages maintain a low level of activity, and the T_reg_/T_h_17 ratio favors T_regs_. During homeostasis, potential pathogens such as Enterotoxigenic *Bacteroides fragilis* (ETBF) are present as commensal organisms amongst the normal flora of the gastrointestinal tract. (**B**) Unknown environmental signals can activate secretion of a metalloprotease enterotoxin called *Bacteroides fragilis* toxin, or BFT, leading to loss of commensalism and damage of the colonic epithelial barrier through enzymatic cleavage of epithelial tight junctions. *B. fragilis* neuraminidase [24] breaks down the mucus layer to allow deeper penetration of both BFT and the pathogen itself. Invasion of the lamina propria triggers the activation of dendritic cells and shifts the balance of T_reg_/T_h_17 to now favor T_h_17 cells and an inflammatory response. Breach of the intestinal barrier can lead to secretory diarrhea, inflammatory colitis, and extraintestinal infections. For clarity, B cells, ILCs, and other potentially relevant immune cells have been omitted.

Physical containment of commensal bacteria to prevent unrestricted access to deeper tissue immune inductive sites is accomplished by mucus layers which overlay a single cell layer of IECs covering the lamina propria, where other immune inductive cells reside [14]. A thin layer of mucus covers the small intestinal epithelial tissue, while two distinct layers of mucus cover the epithelial tissue of the colon. These layers of mucus serve to physically sequester commensal organisms from colonizing and activating cytokine signaling from IECs (Figure 1A); the mucus also traps both secretory IgA and antimicrobial peptides secreted by IECs to prevent microbial overgrowth and maintain homeostasis [15]. The underlying IECs function as bidirectional gate keepers that transmit induction signals to underlying lamina propria innate immune cells and subsequently transport secretory IgA (SIgA) via polymeric immunoglobulin receptors (pIgRs) out into the mucus bed and intestinal lumen [22]. Secretion of SIgA can involve T-independent homeostatic responses, where the SIgA comprises low-affinity polyreactive antibodies that bind to a variety of commensal organism structures, including lipopolysaccharides, flagellin, and capsular polysaccharides; the mechanism by which these polyreactive antibodies are generated remains unknown [23]. T-dependent adaptive responses produce conventional high-affinity and antigen-specific antibodies generated in regional lymph node germinal centers by induction mechanisms resembling systemic responses [23]. Numerous potential biological functions for SIgA have been proposed, including immune exclusion of bacteria through entrapment in mucus and interference with motility, although scant in vivo data exist in support of any of these mechanisms.

The balance between commensal homeostasis and the induction of inflammatory reactions, accompanied by subsequent adaptive responses against pathogenic organisms, depends on the ratio of CD4^+^ Foxp3^+^ regulatory T cells (T_regs_) to stimulatory CD4^+^ RORγt^+^ helper T cells (Th17 cells) [25,26,27,28] and is strongly influenced by local antigen-presenting cells (APCs), including both resident macrophages and dendritic cells (Figure 1A). Resident macrophages in the lamina propria that are intimately associated with IECs are typically non-inflammatory, non-migratory cells that respond to opportunistic invasion by non-pathogenic bacteria via phagocytosis and intracellular killing [16,29,30,31]. Different from other macrophages, these macrophages have low levels of surface toll-like receptors (TLRs) that normally respond to pathogen-associated microbial patterns (PAMPs) to elicit activation and secretion of inflammatory cytokines; therefore, non-inflammatory resident macrophages that phagocytose commensal bacteria do not become activated, but rather secrete IL10, which stabilizes surrounding tolerogenic T_regs_ [32]. Dendritic cells residing in the lamina propria can also respond to opportunistic non-pathogenic bacteria, perhaps due to local damage of the epithelial barrier. In this case, however, dendritic cells are able to migrate from the lamina propria to regional lymphoid tissue, directly presenting antigen to B cells in a T-independent manner to stimulate secretion of low-affinity polyreactive SIgA antibody into the mucus [33] to bind to and immobilize commensal bacteria, and thereby prevent further breach of the intestinal barrier [25,34]. In mice, IgA-coated commensal organisms can be taken up by M cells in the Peyer’s patches of the small intestine and presented to underlying dendritic cells to elicit production of immunosuppressive IL-10 and stimulation of T_regs_, thereby maintaining antigen-specific, commensal-targeted homeostasis [35,36]. Non-activated dendritic cells migrating to regional lymph nodes also secrete retinoic acid and TGF-β, leading to differentiation of naïve T cells into tolerogenic T_regs_ expressing gut-homing α_4_β_7_ and CCR9 surface markers that facilitate maintenance of gut homeostasis [28,31,37,38]. These T_regs_ secrete TGF-β and IL-10 to suppress Th17 cells and inflammation [26,39].

Despite the central importance of APCs in maintaining homeostasis versus adaptive immunity, there are several other subsets of intestinal cells that are important in distinguishing between non-pathogenic and pathogenic organisms. Perhaps not surprisingly, IECs play an important role in sensing the invasion of microbes. Flagellin appears to be one important signaling molecule which binds to TLR5. Importantly, TLR5 is expressed in the basolateral membrane of non-activated IECs rather than in the apical membrane, where the concentration of flagellin from commensal organisms would be highest [33,40,41]. This compartmentalization of TLRs ensures that activation of IECs will likely occur from invading organisms coming into contact with the basolateral membrane of IECs (rather than from incidental contact with commensal organisms), resulting in secretion of proinflammatory cytokines as well as antimicrobial peptides. Interestingly, TLR2 (activated by lipoprotein ligands) is expressed on the apical side of IECs [22] and may therefore encounter ligands from commensal bacteria; however, TLR2/MyD88 signaling of IECs induces the generation of T_reg_s [21], again avoiding unnecessary and potentially damaging inflammatory responses. Potentially pathogenic invading organisms which successfully penetrate the IEC barrier encounter underlying dendritic cells, resulting in migration of fully activated dendritic cells to regional lymph nodes to elicit T cell-dependent adaptive responses through induction of helper T cells, including Th17 cells, to tip the balance away from tolerance dominated by T_regs_ [42,43].

Another population of immune cells that play an important role in the maintenance of homeostasis are innate lymphoid cells (ILCs), which do not express high-affinity antigen receptors [44]. ILC3s, an important subset of ILCs typically associated with IECs, are activated by secretion of IL-23 from dendritic cells that have been stimulated by commensal bacteria [45]. Activation of ILC3s induces secretion of IL-22, which, in turn, stimulates IECs to secrete antimicrobial peptides into the mucus as well as stimulating the repair of tight junctions to mitigate any potential breaches to the epithelial barrier [16,22,45]. In this way, commensal organisms are again prevented from penetration into deeper tissues to stimulate unnecessary adaptive immune responses. In the absence of epithelial tissue damage, IECs secrete IL-25, which suppresses ILC3-mediated release of IL-22 to prevent overexpression of antimicrobial peptides [45,46].

It is clear then that the maintenance of a symbiotic relationship with non-pathogenic bacteria in the gastrointestinal tract is preserved by intricate pathways of cytokine communication between IECs, ILCs, macrophages, and dendritic cells, with redundancy built in to ensure proper segregation of normal flora from immune inductive sites. Breach of these barriers by pathogenic organisms, equipped with an array of virulence factors capable of defeating primary defenses against infection, triggers innate inflammatory responses and full activation of dendritic cells, which migrate to regional lymph nodes to elicit robust induction of adaptive immunity.

## 4. Why Are Reactogenic Vaccines Highly Immunogenic?

In view of the preceding discussion, it becomes clear that one reason that pathogenic organisms trigger pathogen-specific adaptive immune responses involves the activation of inflammatory responses, which recruit innate immune cells such as systemic macrophages, neutrophils, and cytotoxic natural killer cells to the area of infection to rapidly begin phagocytosing and killing invading organisms while activating other antigen-presenting cells such as dendritic cells. The mechanisms controlling the level of immune response against a given bacterial organism depend on overlapping and complementary detection strategies capable of discriminating between non-pathogenic and pathogenic organisms to mount either a tolerogenic non-inflammatory response or to trigger more robust adaptive immunity, respectively. Indeed, a theory describing a series of immune checkpoints for scaling the threat of a microbial infringement has recently been advanced [47,48], for which some formal evidence in support of specific aspects of the theory is now accumulating.

These immune checkpoints can respond to five key properties of invading pathogens that have been postulated to influence the strength of an adaptive immune response through interaction with these checkpoints. The first property concerns the particulate nature of ligands associated with invading pathogens, which is proposed to signal a higher microbial threat to the host immune system than soluble ligands. This would imply that intact organisms possessing multiple cell-associated ligands to activate different pattern-recognition receptors (such as lipopolysaccharide activation of TLR4 or flagellin activation of TLR5) could result in robust activation of innate immune cells and subsequent pathogen-specific adaptive immunity by multiple signaling pathways [49]. Intact pathogens could also present a higher density of surface antigens directly to B cells, thereby eliciting higher antigen-specific antibody responses than soluble antigens [50]. Support for this mechanism of particulate antigens stimulating robust antigen-specific immunity has recently been reported by Kanekiyo et al. [51], who used synthetic nanoparticle-based vaccines to elicit protective immunity against the Epstein–Barr virus in a mouse model. These nanoparticles presented symmetrically arrayed copies of the cell-binding ectodomain of gp350 (the cell-binding surface glycoprotein of the virion) that elicited virus-specific neutralizing antibody titers 10–100 fold higher than levels raised by soluble antigen, and significantly protected against challenge [51]. Additional evidence has been reported by Micoli et al. [52], who compared humoral immunity in mice against lipopolysaccharide O-antigens from *Salmonella enterica* serovar Typhimurium presented to the immune system either as particulate purified outer membrane vesicles (OMVs) or as soluble conjugate vaccines in which the O-antigen was chemically conjugated to the hapten carrier protein CRM197. Particulate OMVs were able to induce higher O-antigen-specific IgG responses with broader IgG subclass and Ig isotype profiles than soluble glycoconjugates, accompanied by strong bactericidal activity and reduced bacterial colonization, in mice challenged with virulent *S.* Typhimurium strains. Importantly, OMVs did not require the use of adjuvants while non-adjuvanted glycoconjugates were not immunogenic [52].

The second property of invading pathogens proposed to affect adaptive immunity is viability. Live organisms elicit more robust immune responses than dead organisms. In this respect, mRNA may be a key signaling molecule for the presence of live microbial threats because it is normally degraded very quickly and is not present in high levels in non-living organisms. In support of this possibility, over a decade ago, it was first observed that the immunogenicity of killed bacteria could be raised to the level elicited by live-attenuated bacteria, both administered parenterally in equal dosing schedules, by supplementation of killed vaccine with purified mRNA [53]. Accumulating evidence in human antigen-presenting cells now suggests that bacterial mRNA can function as a molecular signal of increased threat by invading pathogens through the signaling of endosomal TLR8 and subsequent inflammasome activation [54].

The third property of invading pathogens affecting adaptive immunity concerns live organisms with pathogenic factors facilitating colonization and/or secretion of toxins, which elicit more robust inflammatory and adaptive immune responses than non-pathogenic organisms without these additional factors. Excellent summaries of pathogenic secretion systems and other virulence factors, specifically relating to enteric bacteria*,* have been previously reported and will not be recapitulated here [55,56,57,58]. However, in the case of *Salmonella* (including *S.* Typhi), expression of ancillary effector proteins such as SopB is capable of delaying inflammatory responses by interrupting the proper function of TLRs through interference with adapter proteins such as TIRAP, which is required to facilitate downstream signaling of activation pathways [59,60]. This activity highlights the benefit to *Salmonella* of avoiding early inflammatory responses that could delay or diminish infection and promote clearance by adaptive responses.

The last two properties of invading pathogens affecting immune responses involve invasion of host barriers, frequently initiated when endothelial tissue is breached, which triggers inflammatory reactions. This activity appears to influence the maintenance of non-invasive commensals in the gastrointestinal tract through immune homeostasis, as discussed above. However, deep tissue penetration into normally sterile sites, in which homeostasis does not occur, provides robust signaling for inflammatory and adaptive responses. The impact of these two properties on inflammatory responses is clearly illustrated in the case of gastrointestinal colonization with *Bacteroides fragilis*, an organism that displays both commensal and pathogenic behavior in the same human host (Figure 1A,B). Certain sub-species of *B. fragilis,* designated as Enterotoxigenic *B. fragilis* (ETBF), carry a unique pathogenicity island (BfPAI) encoding a potent metalloprotease enterotoxin called *Bacteroides fragilis* toxin, or BFT [24]. Despite carrying BfPAI, ETBF can be detected as commensal organisms in the colon of healthy individuals displaying no obvious signs of disease [61,62]. It has been demonstrated that the two-component signal transduction system RprX/Y downregulates BFT to maintain intestinal homeostasis and prevent disease [63]. However, unknown environmental signals can activate secretion of BFT, leading to loss of commensalism and damage of the colonic epithelial barrier through enzymatic cleavage of epithelial tight junctions (Figure 1B). The clinical outcome of this transformation includes secretory diarrhea, inflammatory colitis, and extraintestinal infections. 

These five properties of invasive pathogens, proposed to stimulate immune checkpoints controlling innate and adaptive immunity, seem to explain why reactogenic live oral vaccines have historically generated the most highly immunogenic pathogen-specific immune responses, while over-attenuated candidate vaccine strains have not provided biologically relevant immunity. Vaccines able to induce sufficient inflammatory responses via multiple signaling pathways can elicit a robust adaptive immune response capable of conferring protection against disease, while over-attenuated strains may be quickly cleared by innate cells (such as non-inflammatory macrophages) without eliciting inflammation. However, achieving the proper balance between acceptable reactogenicity and protective immunogenicity has proven to be a very elusive goal for engineering appropriately attenuated *Salmonella* vaccines in general and *Salmonella*-based carrier vaccines in particular.

## 5. Balancing Attenuation and Immunogenicity in Carrier Vaccines

In addition to the ability to stimulate innate immunity, other critically important factors influence the quality of an adaptive immune response against carrier vaccines and the foreign antigen(s) that they present to the immune system. Based on data from both murine intranasal animal models [64,65,66] and clinical trials (see Table 1), the factors influencing immunity can be categorized into four main groups whose interactive relationships combine to collectively affect the type, specificity, and magnitude of the immune response (Table 2). Given that a number of different strategies can be either engineered or carefully chosen to minimize any detrimental influence that each of these factors may exert on carrier vaccine immunogenicity, we will focus our discussion here on key concepts, and refer the reader to excellent reviews published elsewhere for more detailed discussions of approaches that have shown significant promise in animal models for improving responses in clinical trials.

### 5.1. Parental Strain Factors

We hypothesize that retention of as many native characteristics of a given pathogen as possible, to elicit robust carrier and foreign-antigen-specific immunity while avoiding over-stimulation of unacceptably reactogenic inflammatory responses, is essential to the development of an immunogenic carrier vaccine. In the case of *S.* Typhi-derived carrier vaccines, we reason that limited invasion of gastrointestinal tissue, facilitated by retention of multiple virulence factors of *S.* Typhi, will result in the vaccine reaching immune inductive sites in numbers sufficient to trigger an innate immune response that triggers adaptive immune responses, without high levels of replicating organisms precipitating more serious inflammatory reactions. This delicate balance between minimal reactogenicity through attenuation and robust immunogenicity can be achieved through chromosomal deletion of two types of genetic loci: 1) deletion of critical genes with pleiotropic effects on the expression of multiple factors essential to survival within the host, or 2) deletions which interrupt metabolic pathways to restrict the replication of vaccines within the host. Examples of carrier vaccines in which pleiotropic deletion mutations resulted in clinically acceptable attenuated vaccine candidates include deletions of *phoP/phoQ* (Ty1033, [8]), *ssaV* (TSB7, [11]), and *htrA* (CVD 908-*htrA*; [7,67,68]); all of these attenuated strains can no longer establish systemic infections within the host, which eventually leads to the classic pyrogenic state typical of typhoid fever. Examples of attenuating strategies which compromise the metabolic fitness of the candidate vaccine strain include deletion of Δ*aroC*Δ*aroD* (CVD 908, [12,69]) and Δ*cya*Δ*crp* (“chi strains”, [13,70]). In this regard, it should be emphasized that vaccine strains attenuated through compromised metabolic pathways maintain a full complement of virulence genes, which could result in unacceptable systemic infection with insufficiently attenuated vaccine candidates. This point is illustrated in the development of the vaccine strain CVD 908-*htrA*, in which the first iteration of the vaccine, CVD 908, was attenuated only with the deletions of *aroC* and *aroD*, resulting in the inability to synthesize aromatic amino acids and purines. Clinical trials with CVD 908 resulted in the detection of vaccine organisms in the blood (vaccinemia) of volunteers receiving a single oral dose containing 10^7^ viable organisms [67]; introduction of the further deletion of *htrA*, a stress protein, prevented vaccinemia while maintaining robust humoral and cellular immune responses against the vaccine strain [67,68].

In contrast to under-attenuated vaccine strains, over-attenuation of vaccine strains can result in candidates that prove completely safe and non-reactogenic in subjects but also fail to elicit immunogenic responses to either the carrier itself or the foreign antigen [71]. However, in some cases, this drop in immunity can be overcome by increasing the number of doses administered. This point is clearly illustrated in clinical trials of carrier vaccines derived from CVD 908-*htrA* and Ty21a. Ty21a is a licensed typhoid live vaccine derived from the wild-type clinical isolate Ty2 by treatment with a chemical mutagen, resulting in multiple attenuating chromosomal lesions [72,73]; in contrast, CVD 908-*htrA* is a genetically engineered vaccine candidate in which only three precise deletion mutations have been introduced into Ty2 [67]. In clinical trials of CVD 908-*htrA* and Ty21a carrier vaccines expressing the urease foreign antigen from *Helicobacter pylori*, three doses of the Ty21a carrier containing 10^10^ viable organisms were required to elicit urease-specific humoral immunity comparable to levels elicited by CVD 908-*htrA* administered in a single dose of 10^8^ viable organisms [12]. These data suggest that in cases where the carrier strain may be over-attenuated and elicits insufficient immunity after a single dose, foreign-antigen-specific immunogenicity can be improved either by increasing the number of doses administered or by boosting with adjuvanted antigen. However, in cases where expression of the foreign antigen proves toxic to an already optimally attenuated and clinically acceptable vaccine strain, increasing the number of doses administered is not likely to overcome such a severe drop in fitness. The resulting over-attenuation would result in rapid clearance of vaccine organisms, insufficient activation of innate and humoral immunity, and insufficient levels of foreign antigen ultimately being delivered to the adaptive immune system to elicit antigen-specific immunity [71]. 

### 5.2. Antigen Expression Factors

Since the first clinical trials conducted with carrier vaccines in the 1980s [2,3,4], significant efforts have been invested in technologies for optimizing the expression levels of foreign antigens to stimulate biologically relevant immunity, including either mucosal, humoral, or cellular immune responses [71,74,75,76,77]. It is well appreciated that carrier vaccines derived from attenuated *S.* Typhi are fully capable of eliciting mucosal, humoral, and cellular responses after oral immunization in humans [78,79,80,81]. However, the design of carrier vaccines presents unique challenges posed directly by expression of the foreign antigen that are not encountered in the engineering of attenuated parental vaccines per se. In addition to the potential toxicity of a given foreign antigen when expressed in a carrier vaccine, which can directly interfere with the viability of the strain [82,83], inappropriate expression of high levels of an otherwise tolerable foreign antigen can stress the metabolic fitness of a carrier vaccine [84,85,86,87]. We have written extensively on this topic and will not recapitulate detailed arguments herein, but rather refer the reader to more extensive discussions of mechanisms published elsewhere [71,74,75]. Here, we emphasize that inappropriately high expression levels of foreign antigens create a physiological burden on a vaccine strain. This reduction in fitness is functionally equivalent to over-attenuation of the vaccine strain, reducing both the replication of these organisms and their ability to reach immune inductive sites at sufficient levels to elicit both innate and adaptive immunity.

Over-attenuation of carrier vaccines due to the metabolic burden associated with inappropriate expression of foreign antigens often occurs when these antigens are expressed from plasmids of high copy number [88]. This potential problem can be overcome both by varying the strength of the promoter controlling transcription of the foreign gene, as well as using tightly regulated plasmids with inherently low copy numbers. A particularly elegant choice of regulated promoters involves the use of promoters which are activated in the presence of environmental signals likely to be encountered in the host after oral immunization, such as osmolarity [88], oxygen tension [89], and environmental shock [89]. However, plasmid-based expression of foreign antigens exerts metabolic pressure on the carrier vaccine that can induce the spontaneous loss of the plasmid in vivo to improve growth rate. For this reason, “balanced lethal” strategies have been employed to prevent spontaneous plasmid loss which would obliterate foreign antigen-specific immunity [90,91]. Of note, these balanced lethal systems do not rely on the use of antibiotic resistance genes for introduction of expression plasmids into carrier vaccines, which also avoids the unintended spread of such resistance genes to other gastrointestinal organisms. The strategy behind balanced lethal plasmid retention involves essential factors encoded for on the expression plasmid which have been deleted from the bacterial chromosome; therefore, loss of the plasmid destroys the ability to synthesize the essential factor required for growth and the plasmid-less vaccine organisms are removed from the growing population. To date, balanced lethal systems based on *thyA* [5,9,10,12], *asd* [1,13]*,* and *purB* [8] have been evaluated in clinical trials with varying degrees of success. To avoid both plasmid instability issues and overexpression of foreign antigens, investigators have also chosen to integrate foreign gene cassettes into the chromosome of the carrier vaccine, again with varying degrees of success in clinical trials [6,11]; the drop in gene dosage of the foreign gene can result in a further drop in the level of foreign antigen synthesized and presented to the immune system, again leading to carrier vaccine failure.

Yet another issue that must be grappled with is the stability of the foreign antigen once synthesized by the carrier vaccine. Data from murine models of immunogenicity strongly suggest that surface expressed antigen, or antigen secreted from a *Salmonella*-based carrier vaccine into the surrounding milieu, is significantly more immunogenic than cytoplasmically expressed antigen that may aggregate into insoluble inclusion bodies or be degraded by proteases [71]. A number of promising antigen export systems have been tested in animal models [92,93,94,95,96,97,98,99,100], but to date, only one clinical trial has been conducted in which the foreign antigen was designed to be secreted [13]. Although exported from multicopy expression plasmids, volunteers failed to mount antigen-specific antibody responses against *Streptococcus pneumonia* surface protein A (PspA). The authors of this study speculated that poor growth of the vaccine in vivo, perhaps due to hyperattenuation, may not have permitted sufficient synthesis of PspA to stimulate immunity, again emphasizing the strict requirement for the carrier vaccine to reach immune inductive sites in sufficient numbers to elicit biologically relevant immune responses [13].

Supposing that one is able to adjust stable but sufficient expression levels of a foreign antigen while avoiding over-attenuation, an additional property impacting on antigen-specific immunity is the inherent immunogenicity of the antigen itself. This parameter is clearly observed in clinical trials of carrier vaccines expressing either the urease virulence factor from *Helicobacter pylori* [9,10] or a fusion protein of the outer membrane proteins OprF and OprI from *Pseudomonas aeruginosa* [12]. Recognizing the inherent uncertainty associated with comparing results from independently conducted clinical trials, we note that the same licensed vaccine Ty21a was used as the carrier strain in both studies. Both UreAB and OprF-OprI proteins were expressed from similar *thyA*-stabilized expression plasmids with medium copy numbers as well, and subjects received three oral doses of ~10^10^ viable organisms per dose in both studies. However, for subjects immunized with Ty21a expressing UreAB, 0/9 volunteers developed antigen-specific IgG responses, although rises in cell-mediated immunity were noted. In contrast, 15/16 volunteers receiving Ty21a expressing OprF-OprI seroconverted. To further emphasize this point, 13/16 volunteers seroconverted after receiving three doses of 100-fold less viable CVD 908-*htrA* organisms (i.e., 10^8^ viable organisms per dose) and developed antibodies against OprF-OprI expressed from the identical *thyA*-stabilized expression plasmid in the same study [12]. These observations underscore the notion that despite an optimally engineered carrier vaccine, choice of the target foreign antigen remains crucial to the ultimate success of the vaccine, despite the number of doses administered.

### 5.3. Immunization Strategies

Clinical trials have proven essential for working out an effective immunization strategy, capable of eliciting robust and biologically relevant immunity against foreign antigens delivered to the immune system after oral inoculation. Of the trials conducted to date, both single and multiple dosing schedules have been tested (Table 1), with several important observations becoming clear from these studies. Contrary to data from mice orally immunized with *Salmonella*-based carrier vaccines [101,102,103,104], repeated oral immunization of humans does not reduce foreign-antigen-specific immune responses due to immunity elicited against the *S.* Typhi-based carrier strain itself, which could theoretically reduce the numbers of organisms reaching immune inductive sites. Indeed, in studies conducted by T.F. Meyer and colleagues [9,10], it was shown that priming subjects with the empty vaccine strain itself actually improved foreign-antigen-specific cellular immune responses elicited after oral boosting with the carrier vaccine expressing the targeted foreign protein. It has been repeatedly demonstrated in clinical trials that *S.* Typhi-specific immunity elicited by repeated dosing of volunteers with a carrier vaccine has minimal effects on foreign-antigen-specific immunity, even in cases where doses as high as 10^10^ viable counts are given. This observation also appears to be independent of the spacing between doses, wherein neither administration of three doses within an 8-day period [2,3,4,5,6,9,10,12] or two doses separated by up to 56 days [1,11] exerted any effect on *Salmonella*-specific immunity; therefore, the strategy of multiple dosing to elicit optimum humoral or cellular immunity against foreign antigens appears to constitute a sound immunization strategy. This strategy becomes especially powerful in the context of first immunizing orally with one or more doses of *S.* Typhi carrier vaccine and then boosting parenterally with purified adjuvanted foreign antigen, an immunization regime referred to as heterologous prime-boosting and first explored for the improvement of malaria and HIV subunit vaccines [105,106]. Heterologous prime–boosting using *S.* Typhi carrier vaccines has been shown in murine intranasal models of immunogenicity to elicit excellent antigen-specific immunity, often superior to immunity elicited from multiple doses of carrier vaccine alone [100,107,108,109,110]. The success of this novel immunization strategy has also been confirmed in clinical trials [12], with excellent humoral immunity raised against the targeted foreign protein.

### 5.4. Host Factors

In addition to factors influencing the antigen-specific immunity of carrier vaccines, one must also consider factors which can significantly affect the immunocompetence of the population intended to receive a given carrier vaccine. Given the complexity of mechanisms influencing immune function, a number of host-specific determinants should also be expected to affect the quality and specificity of an immune response to a carrier vaccine. Although the age of the individual and comorbidity status are two important determinants currently receiving significant attention (reviewed in [111,112,113,114], respectively), a thorough examination of these and other equally important determinants is beyond the scope of the current discussion. Rather, we limit our discussion to points relevant to the gastrointestinal tract and relating back to our analysis above of homeostasis.

It is now recognized that the immunogenicity of oral viral and bacterial vaccines administered to subjects in developing countries is significantly lower than immune responses observed for identical vaccines given in industrialized countries [115,116,117]. In low-income countries with unsanitary living conditions, people are often chronically exposed to contaminated food and water. As a result, adults and, in particular, malnourished children experience bacterial overgrowth of fecal organisms in the small intestine that are normally restricted to the colon of healthy individuals [116,118]. This leads to chronic histological changes in the small intestines, including blunting of villi and a prolonged inflammatory state that disrupts homeostasis, a condition referred to as environmental enteropathy [119,120]. The presence of fecal organisms outcompeting commensals in the small intestine can lead to a disruption of the intestinal mucosal barrier and systemic penetration of normal microbiota accompanied by endotoxemia [117].

The effects of environmental enteropathy on the immunogenicity of oral bacterial vaccines have been studied most extensively with oral cholera vaccines in clinical trials conducted in both developing and industrialized countries using the identical immunization protocols [121,122,123]. Landmark studies conducted with the live-attenuated cholera vaccine CVD 103-HgR clearly established that seroconversion rates after administration of a single oral dose were significantly higher in industrialized countries of North America and Europe than those observed in developing countries such as Peru, Chile, and Indonesia. It was noted that when the oral dose of viable organisms was increased 10-fold, seroconversion rates in developing countries significantly improved and approached rates seen with lower doses in industrialized populations [121]. A related phenomenon of lower reactogenicity of live vaccines in developing countries versus unacceptable reactogenicity observed with identical vaccines in industrialized settings was noted by Levine [116] with live-attenuated *Shigella* vaccines. In this study, low doses of attenuated organisms were required to avoid reactogenicity while still eliciting protective responses against experimental challenge in North American volunteers; when doses several logs higher were given to subjects in endemic regions of developing countries, neither reactogenicity nor immunogenicity were observed [116].

A leading hypothesis to explain this observation is that the mechanism behind reduced reactogenicity and immunogenicity of oral vaccines in developing countries relates to the chronic pro-inflammatory state of vaccinees, in which preexisting gut inflammation immediately clears incoming vaccine organisms, thereby preventing a sufficient number of organisms from reaching immune inductive sites [116]. It has also been hypothesized that the effect of fecal organisms reaching the blood of individuals leads to suppression of dendritic cells (antigen-presenting cells) and thereby lowering of the adaptive immune response [116,117]. Evidence for this hypothesis was reported by Hughes et al. [124], who documented reduced T cell activity and defects in dendritic cell activation in children that were linked to endotoxemia, accompanied by elevated levels of immunosuppressive IL-10; these “anergic” dendritic cells failed to support T cell proliferation, which would be expected to reduce adaptive immune responses to oral vaccination. Interestingly, as noted by Levine [116], these immunosuppressive effects in low-income regions are not observed with the live oral typhoid vaccine Ty21a. Levine hypothesized that the robust immunity elicited by Ty21a might be due to the unique ability of *S.* Typhi to quickly penetrate the gastrointestinal barrier and reach critical gut-associated lymphoid tissue to stimulate both innate and adaptive immunity. This potential characteristic of *S.* Typhi-based vaccines lends support to our previously stated proposition that the most immunogenic live-attenuated carrier vaccines will carry the lowest number of deleted virulence determinants, while at the same time conferring maximum safety and minimum reactogenicity to a clinically acceptable candidate vaccine. As we have pointed out in our discussion above, this balance can be achieved by strategic deletion of genes controlling essential metabolic pathways, the interruption of which prevents the synthesis of high enough levels of virulence determinants at the most permissive anatomical sites to circumvent immune surveillance and cause disease.

## 6. Conclusions and Future Directions

We have explored the current state of carrier vaccine performance in clinical trials and have further discussed the influence of four key factors in determining immune responses against both the carrier strain itself and, perhaps more importantly, the foreign antigen (Table 1 and Table 2). Although we have explored each of these factors independently, it is clear that the interrelationship between each factor will also affect the immunological outcome of oral vaccination with carriers. The importance of innate immunity was also highlighted as being important in eliciting a robust antigen-specific adaptive immune response, although the strength of the inflammatory response must not be overly strong so as to trigger unacceptable clinical reactions.

The clinical trials completed thus far have each provided important pieces of information essential to solving the puzzle of how to engineer and successfully test a highly immunogenic carrier vaccine against an unrelated human pathogen. Arguably, the most successful clinical trial conducted to date was reported by Bumann et al. [12], in which oral immunization with both licensed and experimental carrier vaccines compared favorably to parenteral immunization using multiple doses of an adjuvanted subunit vaccine. This important clinical trial took advantage of many optimized parameters for the factors (described above) affecting carrier vaccine immunogenicity, including:

(1) the choice of an attenuated *S.* Typhi parental strain that did not become over-attenuated upon expression of a foreign antigen and required only one dose for priming (parental strain factor);

(2) expression from genetically stabilized multicopy expression plasmids of sufficient foreign antigen to elicit biologically relevant immunity (antigen expression factors);

(3) use of a heterologous prime–boost strategy in which a single dose of carrier was followed by a single dose of adjuvanted subunit vaccine to elicit robust immunity (immunization factor);

(4) immunization of healthy North American adult volunteers (host factors).

We recognize that the use of North American volunteers may not be predictive of outcomes expected after oral delivery of candidate carrier vaccines in developing countries. This is possibly due to environmental enteropathy and therefore might only be considered as a “best possible case” for human immune responses when compared to target populations in endemic regions for which a given vaccine is intended. 

Despite the promise of this clinical trial, several other considerations should be examined in the future to improve the utility of the carrier vaccine approach. Either secretion or surface expression of the foreign antigen should be explicitly examined in optimized carrier strains to further improve antigen-specific immunity. In addition, most previous trials have only examined humoral responses against the foreign antigen, although several trials reported by T.F. Meyer and colleagues [9,10] confirmed that oral priming with empty carrier strain followed by oral boosting with antigen-expressing carrier elicited significant cellular immunity against the foreign antigen. Therefore, a systematic examination of both humoral and cellular immunity against foreign protein targets could significantly advance the successful development of more immunogenic and protective carrier vaccines. Finally, no trial conducted to date has ever delivered more than one foreign antigen to the immune system; assuming that presentation of more than one protective antigen could significantly elevate protective efficacy without over-attenuating the carrier, it would be important to evaluate the immunogenicity of such a multivalent vaccine, followed by appropriate challenge, if such a challenge model was available.

Here, we have attempted to highlight promising technologies and approaches that might be exploited to improve the success of *S.* Typhi-based carriers as human vaccines. Although experimental animal models have proven essential to the initial design and pre-clinical testing of immunogenic carrier vaccines [77,125], it is ultimately human clinical trials that will definitively guide the development of *S.* Typhi-based carrier vaccines capable of eliciting robust protective efficacy against both *S.* Typhi and the unrelated foreign target pathogen as well. We have explored the retention of as many virulence properties of a candidate carrier vaccine as possible to elicit sufficient innate responses leading to robust adaptive immunity. However, we note that the strategies discussed here are not limited to enteric pathogens per se. These approaches can also be applied to non-pathogenic bacteria engineered as carrier vaccines; we refer the reader to other excellent reviews for a more in-depth examination of engineered non-pathogenic carrier vaccines [126,127]. Regardless of the type of carrier vaccine used, we look forward, with great optimism, to future results from well-designed clinical trials testing optimized carrier vaccines that incorporate the latest in genetic stabilization and gene expression technologies.

## Figures and Tables

**Table 1:** Clinical trials of oral immunization with attenuated *Salmonella enterica* serovar Typhi attenuated carrier vaccines expressing foreign (heterologous) antigens.

Vaccine **^A^**	Year(s) of Clinical Trial	Parent Strain; Relevant Genotype	Foreign Antigen	Expression (Stabilization) Method *^‡^*	CFU Per Dose; Number of Doses **^#^**	Foreign Antigen—Specific Igg Sero- Conversion Rates	Heterologous Booster Given? (Route) **^B^**	Ref
5076-1C w/ plasmid	1984, 1987, 1990	Ty21a; Str ^R^	*Shigella sonnei* O-antigen	P (ND)	~10^9^; 3 doses	28/77	NO	[2,3,4]
EX645 w/ plasmid	1990	Ty21a; Δ*thyA rfa*^Ty2^::*rfa^E. coli^* Rif^R^	O-antigen from *Vibrio cholera* O1 serotype Inaba	P (*thyA*)	~10^10^; 3 doses	1/14	NO	[5]
CVD 908	1994	Ty2; Δ*rpoS* Δ*aroC* Δ*aroD*	*csp* **^1^**	C (NR)	5 × 10^7^; 2 doses	2/10	NO	[6]
χ4632(pYA3167)	1996	Ty2; Δ*cya* Δ(*crp-cdt*) Δ*asd*	HBcAg-pre-S1-pre-S2 **^2^**	P (*asd*)	3 × 10^9^; 3 doses	0/7	NO	[1]
CVD 908-*htrA* w/ plasmid	2000	Ty2; Δ*rpoS* Δ*aroC* Δ*aroD* Δ*htrA*	*toxC* **^3^**	P (ND)	10^8^ to 10^9^; 1 dose	3/9	NO	[7]
Ty1033 w/ plasmid	2000	Ty2; Δ*rpoS* Δ*phoP* Δ*phoQ* Δ*purB*	*ureA-ureB* **^4^**	P (*purB*)	>10^10^; 1 dose	0/7	NO	[8]
Ty21a(pDB1)	2001, 2004	Ty21a; Δ*thyA*	*ureA-ureB*	P (*thyA*)	~6–9 × 10^9^; 3 doses 1–2 × 10^10^; 3 doses	0/9 0/9	NO	[9,10]
TSB7	2007	Ty2; Δ*rpoS* Δ*aroC*::P*_ssaG_-eltB* Δ*ssaV*	*eltB* **^5^**	C (NR)	~10^8^; 2 doses ~10^9^; 2 doses	7/12 15/22	NO	[11]
CVD 908 w/ plasmid	2010	Ty2; Δ*rpoS* Δ*aroC* Δ*aroD* Δ*thyA*	*oprF-oprI* fusion protein **^6^**	P (*thyA*)	~10^8^; 1 dose	13/16	YES (IM)	[12]
Ty21a w/ plasmid	2010	Ty21a; Δ*thyA*	*oprF-oprI* fusion protein **^6^**	P (*thyA*)	~10^10^; 3 doses	15/16	YES (IM)	[12]
χ9639(pYA4088) **^C^**	2013	Ty2; *rpoS*−	*pspA* **^7^**	P (*asd*)	10^7^–10^10^ in escalating single doses	0/20	NO	[13]
χ9640(pYA4088) **^C^**	2013	Ty2; *rpoS*+	*pspA*	P (*asd*)	10^7^–10^10^ in escalating single doses	0/20	NO	[13]

**^A^** When provided in publication, the specific expression plasmid is listed; **^B^** Subjects primed orally with carrier vaccine and then boosted with purified adjuvanted foreign antigen via the intramuscular (IM, adjuvanted with Al(OH)_3_) route; **^C^** Both 9639 and 9640 carry engineered *araC*-controlled mutations in multiple chromosomal loci, which become attenuating as the carrier vaccine replicates in vivo and diminishes intracellular concentrations of arabinose; **^1^** *Plasmodium falciparum* circumsporozoite surface protein; **^2^** protein fusion of peptide fragments from the hepatitis B virus core protein (HBcAg), pre-S1, and pre-S2 regions of the envelope protein (HBsAg); **^3^** non-toxigenic fragment C of tetanus toxin; **^4^** *Helicobacter pylori* urease subunits A and B. Three of these volunteers orally boosted 15 days post carrier vaccine prime with 60 mg recombinant urease plus 2.5 g native purified *E. coli* heat labile toxin (LT) adjuvant; **^5^** Enterotoxigenic *Escherichia coli* (ETEC) heat-labile enterotoxin subunit B; **^6^** fusion protein of *Pseudomonas aeruginosa* outer membrane protein F (OprF; residues 190–342) and outer membrane protein I (OprI; residues 21–83); **^7^** *Streptococcus pneumoniae* surface protein A; ***^‡^*** P, plasmid; C, chromosomal integration; ND; not done; NR; not relevant for chromosomally expressed antigens; **^#^** colony forming units (CFU) per dose received by volunteer in designated number of doses administered orally. R: Resistance.

**Table 2:** Factors potentially affecting foreign-antigen-specific immunogenicity of attenuated *S.* Typhi-based oral carrier vaccines in humans.

Factor	Potential Problem
1. Over-attenuation of the carrier strain itself	Reduced colonization/contact with immune inductive sites
2. Ability of carrier to elicit humoral, cellular, and/or mucosal immunity	Failure to elicit biologically relevant foreign-pathogen-specific immunity
3. Expression level of the foreign antigen; chromosomal versus plasmid-based expression	Metabolic burden over-attenuates carrier vaccine
4. Location of the synthesized foreign antigen; cytoplasmic versus surface or exported	Intracellular degradation of antigen(s) reduces in situ delivery to immune inductive sites
5. Inherent immunogenicity of the foreign antigen(s)	Insufficient stimulation of biologically relevant immunity
6. Immunization strategy; dosing, timing, and homologous versus heterologous vaccination	Insufficient stimulation of biologically relevant immunity
7. Host intestinal microbiota	Reduced colonization/contact with immune inductive sites

## Data Availability

Relevant data is found in the specific references mentioned.
